# Probing Photoreceptor Outer Segment Phagocytosis by the RPE In Vivo: Models and Methodologies

**DOI:** 10.3390/ijms23073661

**Published:** 2022-03-27

**Authors:** Jade A. Vargas, Silvia C. Finnemann

**Affiliations:** Center for Cancer, Genetic Diseases and Gene Regulation, Department of Biological Sciences, Larkin Hall, Fordham University, 441 East Fordham Road, Bronx, NY 10458, USA; jvargas23@fordham.edu

**Keywords:** phagocytosis, phagosomes, RPE, methods, mice

## Abstract

In the vertebrate retina, the light-sensitive photoreceptor rods and cones constantly undergo renewal by generating new portions of the outer segment and shedding their distal, spent tips. The neighboring RPE provides the critical function of engulfing the spent material by phagocytosis. RPE phagocytosis of shed rod outer segment fragments is a circadian process that occurs in a burst of activity shortly after daily light onset with low activity at other times, a rhythm that has been reported for many species and over 50 years. In this review, we compare studies on the rhythm and quantity of RPE phagocytosis using different in vivo model systems and assessment methods. We discuss how measurement methodology impacts the observation and analysis of RPE phagocytosis. Published studies on RPE phagocytosis investigating mice further suggest that differences in genetic background and housing conditions may affect results. Altogether, a comparison between RPE phagocytosis studies performed using differing methodology and strains of the same species is not as straightforward as previously thought.

## 1. Introduction

In the vertebrate eye, lifelong vision is made possible by the intimate relationship between the neural retina and the underlying retinal pigment epithelium (RPE). Photoreceptors maintain their function as the light-sensing cells in the retina through continuous photoreceptor outer segment renewal. This process involves the routine engulfment of shed photoreceptor outer segment fragments (POS) by the RPE. Phagosomes inside the RPE have been observed and analyzed to measure photoreceptor outer segment shedding and renewal for nearly 50 years. This review was driven by our interest in how the methodology and animal model used to assess RPE phagocytosis may affect the comparison between studies.

## 2. Photoreceptor Outer Segment Renewal across Species

Photoreceptor outer segment renewal involves the shedding of spent POS from the photoreceptor distal outer segment tip and simultaneous phagocytosis by the underlying RPE [1,2]. New membrane discs are constantly formed at the proximal end of the outer segment to offset those lost at the distal tip. The molecular mechanisms employed by the RPE to phagocytose POS have been studied for several decades. In the mammalian retina, exposure of the anionic phospholipid phosphatidylserine (PS) by spent POS stimulates initial recognition of spent POS by the RPE [3]. The secreted integrin ligand MFG-E8 (milk fat globule-EGF factor 8) localizes to the subretinal space in between outer segments and RPE to act as a bridging ligand between PS on POS and αvβ5 integrin receptors on the apical surface of RPE [4,5]. Internalization of integrin-tethered POS further requires activation of Mer tyrosine kinase (MerTK) receptors of the RPE, which occurs through direct MerTK ligand activation and cytosolic signaling from αvβ5 integrin binding receptors via focal adhesion kinase [6,7,8]. The number of engulfed POS in phagosomes within the RPE serves as a measure of combined POS shedding/uptake activity and thus photoreceptor renewal.

RPE phagocytosis is a circadian process, with a daily peak of activity shortly after light onset for rod POS clearance that persists in entrained experimental animals even if they are shifted to permanent darkness. Circadian clocks have been identified in both the retina and the RPE, and loss of circadian regulation in mice eliminates the morning peak of RPE phagocytosis (recently comprehensively reviewed by Baba and colleagues [9]). The importance of the daily rhythm of RPE phagocytosis for lifelong retinal health remains uncertain. Recent studies using mouse models lacking key proteins for phagocytosis or rhythm regulation demonstrate no functional or phenotypic abnormalities associated with aging despite a loss of daily rhythmicity [4,10,11]. In contrast, in mice lacking the POS recognition receptor αvβ5 integrin, the lack of the morning peak of phagocytosis is associated with impaired POS digestion and age-related increase in oxidative damage and loss of photoreceptor function [5,12]. Altogether, loss of the morning peak of POS uptake is thus not sufficient to cause retinal dysfunction in mice, and age-related defects may arise in mice lacking αvβ5, for instance, due to additional abnormalities in POS processing. Nonetheless, the daily rhythm of RPE phagocytosis is conserved among all experimental vertebrate species investigated to date, suggesting physiological relevance that may not be obvious in animals maintained under highly controlled conditions. Moreover, it remains a useful metric by which to assess the molecular mechanisms responsible for phagocytosis and its regulation.

Rod photoreceptors constitute the vast majority of photoreceptors in most species explored for experimentation. Rods shed POS for phagocytosis by RPE in the early morning, shortly after light onset. A variety of vertebrate species have been studied to date with similar results. In two different strains of albino rats, Fischer or Sprague-Dawley, the peak of RPE phagosome content is one hour after light onset in rats entrained to a 12-h light/dark cycle [13,14]. Similar to that observed in rats, the RPE phagocytic peak in frogs, chickens, goldfish, and cats occurs shortly after light onset [15,16,17,18,19]. RPE phagocytosis of rods has been observed in numerous mouse strains to date, including C57BL/6, C3H, and 129T2/SvEmsJ [5,10,20,21,22,23,24,25,26]. In all mouse strains studied to date, a peak of rod POS phagocytosis occurs shortly after light onset. Recently, a comprehensive assessment of RPE phagocytosis throughout the day was performed in zebrafish. Peaks in rod POS phagosomes were observed after both light onset and dark onset [26].

In all mammalian species studied, the rhythm of rod shedding and phagocytosis continues when entrained animals are shifted to constant darkness. This persistence indicates that mammalian outer segment renewal is controlled by circadian mechanisms. In contrast, in other vertebrate species like frogs and goldfish, the rhythm does not persist in constant darkness. Of relevance to this review, mammalian rod shedding and phagocytosis by RPE is circadian, and the peak of activity occurs shortly after light onset.

Investigation of cone outer segment renewal is still in its infancy. The timing of cone shedding may be more variable across species than rod shedding but is also diurnal in all species studied to date. It has long been thought that cones tend to have their peak shedding and subsequent phagocytosis in the evening, shortly following the onset of darkness. The cone shedding peak occurs during the dark phase in a number of species where cones are the dominant photoreceptor type. In lizards, chicken, and goldfish, the cone shedding peak is shortly after dark onset, while in the ground squirrel retina, the cones shed in the middle of the dark phase [17,18,27]. However, in cats, tree shrews, and Nile rats, both rod and cone POS phagocytosis peak in the morning after light onset [19,28,29]. This rhythm persists in constant darkness in these species. Notably, while cones make up about one-third of the total photoreceptors in the Nile rat retina, the RPE contains ten times fewer cone POS phagosomes than rod POS phagosomes at peak, suggesting that cones shed less frequently or that cones shed relatively more at off-peak times [30]. Unlike the cone-dominant Nile rat, commonly used mice and rat rodent strains possess retinas where cones make up 3% or less of the total photoreceptors [31,32,33]. Cone POS phagocytosis in these species was largely unstudied prior to the generation of the “all-cone” neural leucine zipper gene (*Nrl*) null mouse *Nrl^−/−^* [34]. Peak cone POS phagocytosis in this mutant mouse, which was generated on a C57BL/6 genetic background, was shown to occur shortly following light onset [35]. The rhythm persisted when mice were shifted to constant darkness. However, retinal abnormalities largely preclude regular use of this knockout mouse for cone outer segment renewal studies [36]. Recently developed methodologies allow for identifying cone POS phagosomes even in retinas where cones represent only a small fraction of total photoreceptors. In C57BL/6 mice, cone POS phagosomes were observed in relatively equal numbers shortly after light and dark onset [26]. We recently reported cone POS phagocytosis in 129T2/SvEmsJ mice, showing 10-times fewer cone POS phagosomes than rod POS phagosomes shortly after light onset at the peak of rod POS phagocytosis [37]. This pattern was also observed earlier in C57BL/6 mice using a different method [38]. Zebrafish retinas are cone-rich, but most studies use the model to specifically observe and measure outer segment disc shedding [39]. However, a recent assessment of phagocytosis in zebrafish revealed light and dark period peaks in cone POS phagocytosis [26].

A summary of phagocytic peak times and methodology used can be found in Table 1. While the previous consensus was that rods shed in the morning and cones shed at night, this does not hold true for all species, and in many species, rods and cones both shed shortly after morning light onset.

## 3. An Intriguing Evening Peak in Rod POS RPE Phagocytosis in Mice

Recently, a previously unnoticed peak of rod POS phagocytosis following dark onset has been reported. This evening peak was reported in both zebrafish and mice [10,26]. For mice, Lewis and colleagues found equivalent numbers of POS phagosomes in the RPE at ZT 1.5 and ZT 13.5, 1.5 h after light onset, and 1.5 h after dark onset, respectively [26]. This study counted POS phagosomes by immunoelectron microscopy of retina sections labeled with rhodopsin antibody B6-30. Goyal and colleagues reported levels of RPE phagosomes at ZT 14 (2 h after dark) that were modestly but statistically significantly higher than off-peak phagosome counts but less than half of the number of RPE phagosomes seen at ZT1 [10]. This study counted POS phagosomes by immunofluorescence microscopy of retina sections labeled with rhodopsin antibody 4D2. Notably, both studies explored mice of the same genetic background, the C57BL/6J strain.

Our studies on the molecular mechanisms of RPE phagocytosis have mostly explored wild-type and mutant mice of 129T2/SvEmsJ strain genetic background. We originally used transmission electron microscopy (TEM) to sample mice every 1.5 h and found that among all time points tested, the number of POS phagosomes in the RPE was greatest at ZT 2, 2 h after light onset as compared to ZT0.5 and ZT3.5 [5]. We did not observe elevated POS phagosome numbers after dark onset measuring ZT 12 (dark onset) and ZT 15 [5]. However, the time points assessed in this study did not include ZT 13.5, the peak of evening phagocytosis seen recently [10,26]. In more recent studies, we mostly sampled POS phagosomes immunolabeled with rhodopsin antibody B6-30, the same antibody used by Lewis and colleagues. However, our morning tissue collection time points were not exactly the same as the time points sampled by the other two recent studies and, again, did not include ZT 13.5 [23,40]. Therefore, it is currently unclear whether the evening peak observed by Lewis and colleagues is present in 129T2/SvEmsJ mice due to differences in sampling times and methodologies employed. Additional studies will be needed to determine whether an evening peak of RPE phagocytosis exists in 129T2/SvEmsJ mice and how it compares to the peak observed in C57BL/6J mice.

## 4. Methodologies Used to Study RPE Phagocytosis In Situ

Intrigued by the different observations of previous studies and current reports, we were curious about how both the RPE phagosome counting methodology and the mouse strain impact the observation and analysis of RPE phagocytosis in situ. The methods and tools utilized for observing and measuring disc shedding and RPE phagocytosis have greatly evolved over the nearly 50 years that this physiological process has remained an intense focus of study. Today, a number of approaches are available and in use.

Light microscopy has long been employed to identify phagosomes within the RPE as it is quick and cost-effective. This method can be used to visualize phagosomes in frozen or paraffin-embedded sagittal retina sections stained with histological dyes. Toluidine blue staining is commonly used in conjunction with light microscopy. Identification of phagosomes is based only on morphological characterization—staining intensity and size. When cross-sections of paraffin-embedded eyecups are stained with toluidine blue, phagosomes present as pale violet inclusions of about 1 µm diameter [13,14,15,18,20,22]. A potentially confounding factor when using light microscopy is the pigmentation of the animal model. Higher numbers of RPE phagosomes have been observed in albino mice than in in pigmented mice [20,21]. Albino animals facilitate more accurate quantification of toluidine blue-stained phagosomes as in pigmented animals, RPE melanosomes may obscure phagosomes [22]. Thus, light microscopy of histological dyes may only be appropriate for assessing phagocytosis in albino animals.

Transmission electron microscopy (TEM) allows for imaging structures within cells via penetration of ultrathin tissue sections ranging from 50 to 90 nm by electrons. TEM has been used extensively to visualize phagosomes within RPE. When using TEM, POS phagosomes inside the RPE can be unequivocally identified by size as well as their morphology, with distinct outer segment discs of POS readily apparent [2,14,17]. Moreover, immuno EM allows added immuno-labeling with POS-specific antibodies and utilizing gold-conjugated secondary antibody to label POS protein [41,42]. However, the high-resolution of images generated by TEM or immuno EM comes at the cost of the breadth of sampling. This concern can be addressed by using toluidine blue staining and light microscopy as a first step to assess broader sections of tissue before focusing on fields for TEM. Despite its advantage of generating high-resolution images, the use of TEM for routine RPE phagosome quantification has declined somewhat over time due to cost and labor considerations.

Recently, a new method was described that combines OCT embedded cryosection immunofluorescence and TEM [43]. This method allows for localization and identification of macromolecules in cryosections, followed by more detailed analysis via EM in the same section. Immunofluorescence staining followed by laser scanning confocal microscopy to identify phagosomes in retina sections allows specific POS phagosome labeling like immuno EM as well as a sampling of large tissue areas.

As an alternative to the extensive tissue processing for embedding and sectioning, the posterior eyecup can be prepared as a whole-mount sample that features exposed RPE after the neural retina is removed. These tissue samples can be live labeled before fixation using organelle dyes or immunolabeled directly without further processing beyond fixation. The resulting RPE whole-mount allows for visualization of the full phagosome content of individual RPE cells [24,37,44,45,46].

Assessing RPE phagocytosis by whole-mount is a powerful tool, but there are both advantages and disadvantages to this method. Analysis of phagosomes in RPE whole-mounts allows for accurate measurement of phagosomes with different sizes. Such size classification can be particularly useful when assessing the maturation state or digestion of phagosomes. The lack of intact outer segments or other neural retinal tissue in this preparation allows for more sensitive visualization of POS phagosomes in the RPE. Moreover, acidified phagosomes in live RPE in freshly dissected posterior eyecup whole-mounts can be readily detected using organelle acidification indicator such as LysoTracker [47,48]. Finally, whole-mount samples allow sampling far larger numbers of RPE per eye and straightforward assessment of regional or cell-to-cell variability in POS phagocytosis in individual eyes.

**Figure 1:**
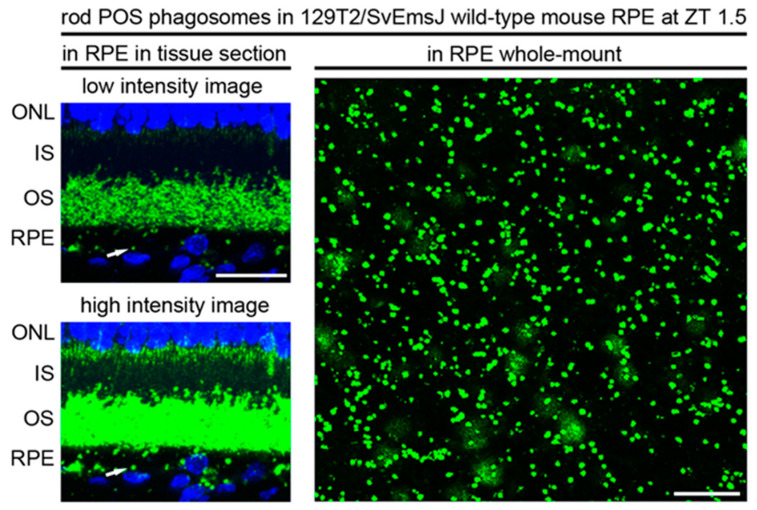
Whole-mount RPE phagosome assessment samples a higher number of RPE per sample than assessment in sagittal sections. Images show 129T2/SvEmsJ tissue obtained at ZT 1.5. Note that detection of RPE phagosomes in tissue sections (left panels) requires high-intensity imaging (lower left), resulting in overexposed intact outer segments (OS). A representative rod POS phagosome is indicated by the arrow. Quantification of high-intensity sections as shown yields a rod POS phagosomes content of 15.3 ± 1 phagosomes per 100 µm length of the central retina on average (mean ± S.D., *n* = 5 eyes from 5 different mice). Imaging the same rhodopsin staining in RPE whole-mount samples (image on the right as indicated) is not confounded by the presence of intact outer segments. Moreover, whole-mount observation allows quantification of the full phagosome load per cell in multiple cells per sample and, thus, importantly, robust assessment of cell-to-cell variability. Quantification of whole-mount images as shown yields 255 ± 29 phagosomes per (100 µm^2^) area of RPE on average (mean ± S.D., *n* = 5). For either quantification method, rhodopsin-positive particles were counted as phagosomes if their diameter was at least 0.5 µm. Scale bars in both fields are 20 µm. Tissue section data are reproduced from Esposito et al., *Invest. Ophthalmol. Vis. Sci.* **2021**, *62*, 7 [40]. RPE whole-mount data are reproduced from Mazzoni et al., *Redox Biol.* **2021**, *42*, 101918 [37].

However, the disadvantages of whole-mount preparation include the inherent tissue disruption that is required. Eyes that are immediately fixed and then embedded require little manipulation and thus undergo minimal disruption of retina-RPE interaction. Whole-mount preparation requires the separation of the neural retina from the posterior eyecup prior to stabilizing fixation, allowing for potential damage and leftover debris. Immunofluorescence labeling of whole-mount tissue has a high animal requirement as one whole eye is required for any one antibody labeling. In contrast, immunofluorescence using sections requires only one eye for potentially quite a number of antibodies, given that a single eye produces a great many sections. Figure 1 shows a direct comparison of rod POS phagosomes in 129T2/SvEmsJ wild-type mouse RPE in tissue sections and in whole-mount both sampled at ZT 1.5 and stained with the same rhodopsin antibody (B6-30), illustrating the greater number of cells sampled using the whole-mount technique and the opportunity to assess absolute phagosome numbers and size distribution per cell in whole-mount but not in section samples.

Altogether, immuno-detection has proven powerful in unequivocally identifying POS phagosomes in the RPE. For any of the approaches involving POS immunolabeling, primary antibodies against rhodopsin have proven most useful, as rhodopsin constitutes the majority of protein in POS and is strictly POS specific [4,49]. In the next section, we will discuss how the selection of POS protein antibodies may affect POS detection.

## 5. Experimental Variables Affecting RPE Phagocytosis Studies: Mouse Strain, Lighting Conditions and POS Protein Antibodies

Genetic differences play a large role in phenotypic differences across the many mouse strains available for experimentation. There are over 200 inbred mouse strains available for use in research studies. In many cases, phenotypic variation among strains can be advantageous, allowing for models that mimic a wide array of human diseases. Over 3000 differentially expressed genes were identified between strains and developmental stages, including those part of major gene networks involved in regulating retinal or photoreceptor detachment, visual perception, and signal transduction [50]. Indeed, the genetic background of mice has been implicated in differences observed in light-induced photoreceptor cell death, injury-induced ganglion cell death, angiogenesis, lymphogenesis, and photoreceptor cell death following retinal detachment [51,52,53,54,55]. Moreover, altered expression of Tyro3, a paralog of MerTK, found in the C57BL/6 and 129P2/Ola (a 129 sub-strain) strains produce markedly different outcomes for photoreceptor cell health [56]. These studies illustrate the variability among wild-type mice from strains of different genetic backgrounds.

C57BL/6 mice are among the most widely used inbred mice. Although this strain was demonstrated to be deficient in melatonin synthesis, direct comparison between C57BL/6 and C3H-f^+/+^, a melatonin-proficient strain, revealed no difference in rod POS phagocytosis rhythmicity [21,57,58]. C57BL/6 mice are commercially available from a number of suppliers and across the world. While convenient and economic access to these mice drives research globally and accelerates overall progress, extensive inbreeding of strains from different sources risks that sub-strains will differ genetically and, eventually, phenotypically. For example, it is extensively documented that the widely available and much-studied sub-strain C57BL6/N (but not the widely used C57BL6/J line) inherently harbor a single nucleotide deletion mutation in the *Crb1* gene, which can greatly confound retina studies [59]. This mutation, known as rd8, causes C57BL6/N mice to develop profound retinal abnormalities with neural retina rosettes involving highly abnormal photoreceptor-RPE interactions [59,60]. Although not directly tested to date, such rd8/rd8 mutant C57BL6 mice can be expected to show abnormal RPE phagocytosis. Notably, a recent screen of commonly studied mouse strains has shown that 129T2/SvEmsJ mice do not harbor the rd8 mutation nor other confounding retinal degeneration (rd) mutations known to date [60]. However, the unexpected discovery of rd8 mutations in wild-type commercially provided C57BL6 mice serves as a cautionary tale, and other mutations affecting the retina or RPE in general and outer segment renewal specifically may be yet to be discovered [61].

In addition to mouse strain differences, animal housing lighting conditions may also affect studies of outer segment renewal. The original purpose of tightly controlled lighting conditions has been to entrain animals to a known circadian rhythm. Defined light and dark cycles of unchanging light intensity allow for precise sampling and comparison between groups with respect to time of day. However, even if entrainment schedules are the same, a comparison of studies performed on the same strain using the same methodology may be confounded if the animal housing light intensities are highly dissimilar. Many (but not all) studies report the lighting conditions in the animal housing and there is a wide degree of variation in light intensity (Table 1). Small differences in light intensity can double phagosome counts, as seen in the ground squirrel [27]. The light intensities used for mice studies are typically around 300 lux. Care must be taken to ensure that no light is entering the animal housing, disrupting the dark cycle, as even brief exposure to light during the dark cycle has the potential to alter the circadian clock and thus affect the timing of RPE phagocytosis. This can be a confounding issue in larger facilities with animal rooms shared among investigators with divergent work schedules and research focus. Allowing time for entrainment to in-house lighting conditions must also be considered when obtaining mice from another facility. While entrainment to phase advance takes several days, entrainment to phase delay takes 3–4 weeks [14,62]. A common practice in our lab is to wait four weeks following any shift in lighting condition before use for circadian studies such as rhythmic RPE phagocytosis. Finally, it should be noted that light intensities in animal studies generally represent indoor, artificial lighting that is much less intense than natural light at sunrise or sunset (~10,000 lux) or midday sunlight (>100,000 lux) [63]. Moreover, light-dark transitions in animal housing are generally abrupt, which does not mimic well day-night changes in a natural environment. Notably, the requirement for light intensity and duration for circadian entrainment differs between mice and human subjects, with mice being far more sensitive, possibly due to their nocturnal nature (for a recent comprehensive review, please see [64]). Nonetheless, adaptive optics assessment of cone POS shedding has found significantly more frequent cone POS shedding at ZT 1 compared to ZT 6 or ZT 12 in healthy adult human subjects entrained to a 16 h on/8 h off light cycle [65]. We conclude that the highly conserved phenomenon of a diurnal rhythm of outer segment renewal set by the day/night light cycle is unlikely an experimental artifact, although the relative effects of circadian regulation, dark-light transition, and abrupt high light exposure remain to be established.

**Figure 2:**
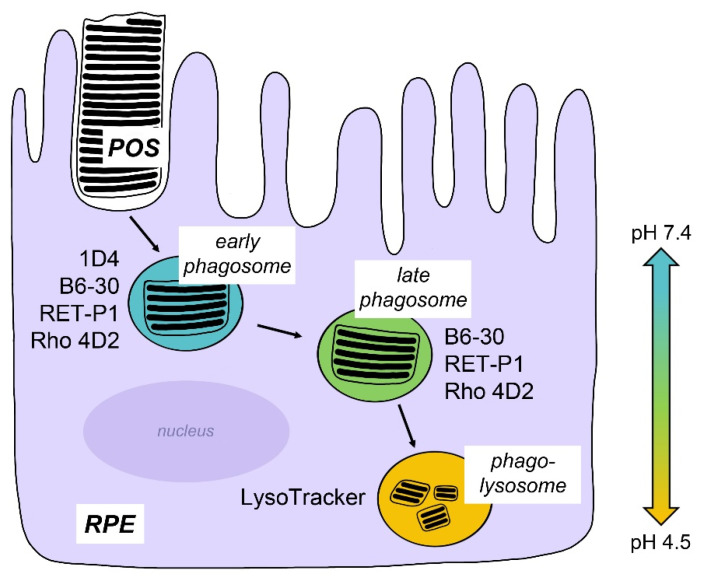
Detection of rod POS phagosomes depends on rhodopsin antibody usage. Freshly engulfed POS in early phagosomes before acidification retain both N-and C-terminal epitopes recognized by commercially available rhodopsin antibodies as indicated. Rhodopsin digestion proceeds in a step-wise fashion with early C-terminus cleavage resulting in loss of epitopes recognized by rhodopsin antibody 1D4 [67,70]. At late stages of phagolysosomal digestion, only LysoTracker labeling provides reliable phagosome labeling.

With respect to technical differences, the choice of POS protein antibody for phagosome labeling plays a particularly crucial role: if the antibody recognizes an antigen that is digested rapidly or that changes conformation (and thus antibody reactivity) in acidic environments like phagolysosomes, the respective antibody labeling will be decreased or even eliminated leading to an undercount of phagocytosed POS. Rhodopsin constitutes the most abundant protein inrod outer segments and thus, rhodopsin antibodies are used commonly to label POS. However, many different monoclonal antibodies to rhodopsin are available. The monoclonal antibody 1D4 recognizes an epitope at the C-terminus of rhodopsin [66]. As engulfed POS phagosomes mature within the RPE, the C-terminus of rhodopsin is rapidly cleaved by an endosome-derived protease [67]. Thus, this antibody is only useful for identifying newly engulfed phagosomes and tracking phagosome-endosome interaction. In contrast, a number of monoclonal antibodies that recognize the N-terminus of rhodopsin are well characterized and commercially available. The monoclonal antibody B6-30 generated and first characterized by the Hargrave lab specifically recognizes the amino acid sequence G3-S14 [68]. The RET-P1 antibody recognizes the first 10 N-terminal amino acids [69]. Rho 4D2 is another commonly used antibody against the N-terminal region of rhodopsin. Antibodies against the N-terminus of rhodopsin are extremely useful as this region of rhodopsin remains intact at first after initial phagosome acidification. The N-terminus with the rest of rhodopsin is also subject to proteolytic degradation eventually, but only after fusion of the mature phagosome with a lysosome. Thus, the N-terminus is the more stable domain compared to the C-terminus and antibodies to N-terminal epitopes allow tracking POS phagosomes until the late stage of phagolysosomal digestion [67,70]. Figure 2 illustrates the changes in the reactivity of widely used, commercially available rhodopsin antibodies with progressing phagolysosomal digestion.

Antibodies against POS proteins other than rod or cone opsin or organelle marker proteins may also be used to assess RPE phagocytosis. Transducin is a photoreceptor phototransduction protein that is not expressed by the RPE and can thus serve as a POS marker [23]. Additionally, RPE protease cathepsin D is essential for opsin digestion and its levels in RPE phagosomes undergo a rhythmicity that parallels that of rhodopsin [23]. Combinations of antibodies may be used to track cone and rod phagosomes in the same RPE samples. Altogether, immunolabeling of POS components is powerful in tracking and specifying the maturation status of engulfed phagosomes in the RPE. However, it is very important to consider how antigen stability will affect POS labeling and thus POS phagosome quantification results. Direct labeling methods such as LysoTracker staining of phagosomes in live RPE preparations ex-situ show many more phagosomes per RPE than antibody labeling post-fixation [47]. Investigating RPE phagocytosis in complementary assays using both live organelle tracker and fixed immunolabeling assays will likely ensure robust results.

## 6. Conclusions

Directly assessing engulfed POS in RPE with the time of day remains a powerful tool for elucidating the molecular mechanisms of POS shedding and RPE phagocytosis. Future studies will be needed to illuminate further and explain the differences of individual experimental models and the consequences of altered photoreceptor renewal for the physiology of the retina.

Comparison between RPE phagocytosis studies performed using differing methodology and strains of the same species is not straightforward due to multiple experimental variables that affect results. Careful consideration of mouse strain, animal entrainment and lighting, times of tissue collection, and methodology for phagosome assessment is crucial for generating data comparable to other studies. Utmost consistency in all of these experimental parameters and their diligent reporting upon publication is critical to ensure data reproducibility.

## Figures and Tables

**Table 1:** Summary of phagocytosis studies in different species and mouse strains.

Species/Strain	Light Entrainment	Light Intensity	Phagocytic Peak	Methodology
Fischer inbred albino rats [13]	12/12 L/D	215–375 lu/m^2^	Rods 0.5–2.25 h after light on	LM
Sprague-Dawley rats [14]	12/12 L/D	10–20 ft-cd	Rods 1 h after light on	LM, TEM
Frog (*Xenopus laevis*) [16]	12/12 L/D	200–250 lx/m^2^	Rods 1 h after light on	LM
Frog (*Rana pipiens*) [15]	14/10 L/D	645 lu/m^2^	Rods 1 h after light on	LM
Chick [17]	12/12 L/D (1 week)	345 lu/m^2^	Rods 1 h after light on; cones 1 h dark	TEM
Goldfish [18]	12/12 L/D (at least 18 days)	700 lux	Rods 1 h after light on; cones 4 h after dark on	LM
Cat [19]	12/12 L/D	270 lux	2 h after light on	LM, TEM
Nile rat (*Arvicanthis ansorgei*) [28]	12/12 L/D	300 lux	Rods/cones 1 h after light on	IF section (Rho 4D2, M opsin)
C57B1 mice [20]	12/12 L/D	200 lux	Rods 2 h after light on	LM
C57BL/6, C3H-f +/+ mice [21]	12/12 L/D	NR	Rods 1 h after light on	LM, TEM
*Nrl^−/−^* C57BL/6 mice [35]	12/12 L/D	300 lux	Cones 1 h after light on	IF section (S opsin)
129T2/SvEmsJ mice [5]	12/12 L/D		Rods 2 h after light on	TEM
C57BL/6J mice [23]	12/12 L/D	NR	Rods 0.5 h after light on	IF section (Rho 4D2)
C3H-f^+/+^ mice [25]	12/12 L/D	NR	Rods 1 h after light on	IF section (B6-30)
C57BL/6J mice [26]	12/12 L/D	NR	Rods/cones 1.5 after light on, 1.5 after dark on	TEM, Immuno EM (B6-30)
Zebrafish [26]	14/10 L/D	NR	Rods/cones 1.5 h after light on, 3.5 after light off	TEM, Immuno EM (anti-rhodopsin)
C57BL/6J mice [10]	12/12 L/D	NR	Rods 1 h after light on, 2 h after dark on	IF section (Rho 4D2)

Abbreviations: NR, not reported; LM, light microscopy; TEM, transmission electron microscopy; IF, immunofluorescence.

## Data Availability

Not applicable.
